# Polarization of Acoustic Waves in Two-Dimensional Phononic Crystals Based on Fused Silica

**DOI:** 10.3390/ma15238315

**Published:** 2022-11-23

**Authors:** Mikhail V. Marunin, Nataliya V. Polikarpova

**Affiliations:** Faculty of Physics Lomonosov, Lomonosov Moscow State University, Moscow 119991, Russia

**Keywords:** two-dimensional phononic crystals, fused silica, finite element method, polarization of acoustic waves

## Abstract

The two-dimensional square-lattice phononic crystal is one of the recently proposed acoustic metamaterials. Strong anisotropic propagation of elastic waves makes the material promising for various potential applications in acoustics and acousto-optics. This paper presents a study of the propagation of elastic waves in two-dimensional phononic crystals based on fused silica. The band structures of a phononic crystal are obtained by solving the wave equation in its variational form by the finite element method. The main phononic crystal acoustic characteristics that are of practical interest in acousto-optics are calculated based on the analysis of the dispersion relations. It is shown that the choice of the phononic crystal geometry makes it possible to control the distributions of both the inverse phase velocities and the energy walk-off angles of acoustic modes. The calculations of the acoustic modes’ polarization are in a particular focus. It is demonstrated that under certain conditions, there are exactly three acoustic modes propagating in a phononic crystal, the averaged polarization vectors of which are mutually orthogonal for any directions of the acoustic wave’s propagation. It is argued that the acoustic properties of phononic crystals meet the requirements of acousto-optics.

## 1. Introduction

Acousto-optic devices are well known for their broad application in research and technology. To date, more than a dozen acousto-optic devices using different principles and serving various objectives have been introduced and explored [1].

In designing new acousto-optic devices, particular emphasis is put on the choice of a material in which optical radiation interacts with an acoustic wave. The choice of material depends on the acoustic, optic, and acousto-optic properties of the medium [2]. Most commonly, priority is given to the acoustic properties of the material because they play a vital role in defining the diffraction efficiency. It is known that the acousto-optic figure of merit of the material is inversely proportional to the cube of the acoustic wave phase velocity [3]. In the acousto-optic field, it is therefore essential to find and analyze the materials characterized by the low phase velocity of propagating acoustic waves.

A crucial factor in choosing the material is the anisotropy of its acoustic properties. Strong acoustic anisotropy makes it possible to create and implement new configurations of acousto-optic interaction. In particular, a high degree of anisotropy of the acoustic wave phase velocity leads to significantly larger values of the acoustic energy walk-off angle. These high values of the angle between the phase and group velocities allow, for example, efficient implementation of the collinear diffraction mode [4,5].

It is known that due to the photoelastic effect, the acoustic perturbation in the medium leads to the induced phase grating in the material. In this case, the polarization vector of the acoustic wave determines the effective photoelastic constant, a value that directly affects the diffraction efficiency [6]. Information about the polarization composition of the acoustic wave is therefore important for practical applications. It is interesting to consider the conditions under which the acoustic wave becomes purely longitudinal or purely transverse, as well as the conditions under which the wave changes the polarization type.

Monocrystalline materials take a special place in the field of acousto-optics. Among the most common and frequently used media are crystals of tellurium (Te), lithium niobate (LiNbO_3_), and TAS (Tl_3_AsSe_3_), as well as various mercury compounds [7,8,9,10]. Nowadays though, a paratellurite (TeO_2_) crystal is used as a working material in acousto-optic devices most of the time.

**Figure 1:**
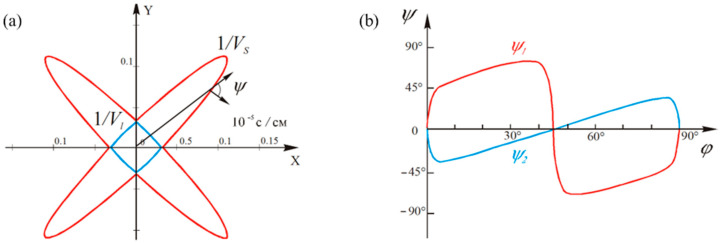
Acoustic characteristics of a paratellurite crystal: (**a**) distributions of the inverse phase velocities of acoustic modes in the XY plane and (**b**) distributions of the walk-off angles of the acoustic modes’ energy in the XY plane.

A paratellurite crystal is known for its unusual acoustic properties, which distinguish it from other monocrystalline media. As an example, Figure 1 shows the spatial distributions of the inverse phase velocities of acoustic waves and their acoustic energy walk-off angles. It is evident that paratellurite has extremely low values of the phase velocity of acoustic waves propagating along specific directions. Thus, the minimum phase velocity of a slow shear acoustic mode propagating along one direction is 616 m/s [11]. To note is the strong acoustic anisotropy of the medium, which leads to record-high angles of acoustic energy walk-off. The maximum value of the acoustic walk-off angle in the paratellurite crystal reaches 74° [12].

A paratellurite crystal is the standard of modern acousto-optics, and it can be used in creating a wide range of acousto-optic devices. However, with a transparency range of 0.35–6.5 μm [13], paratellurite cannot be used in creating acousto-optic devices operating in the ultraviolet and far-infrared regions of the electromagnetic spectrum. Moreover, the physical properties of paratellurite are determined and they cannot be changed, so the value of the acousto-optic effect in the medium is fixed. Thus, an imperative task of acousto-optics today is not merely the search for acousto-optically effective materials but also the development of innovative artificial media, whose properties could be controlled, depending on the unique challenges in creating devices. In this study, we turn to acoustic metamaterials, namely phononic crystals [14,15,16].

A phononic crystal is a periodic structure comprising materials with various acoustic properties [17]. The propagation of acoustic waves in such a crystal is described by the dispersion relation, also known as the band structure. An important feature of the band structure is the presence of band gaps, which are frequency ranges in which the propagation of acoustic waves is forbidden [18]. In addition, under certain conditions, the dispersion curves have a negative slope, which results in negative refraction [19,20]. Moreover, the two-dimensional structures may be applied for tunable broadband polarization converters [21], cloaking sensors [22], and plasmonic photodetectors [23]. The characteristics of phononic crystals are key in studying these materials. However, the periodic nature of phononic crystals predetermines a number of other features, such as strong acoustic anisotropy of the material, low phase velocities of acoustic waves, and large angles of acoustic walk-off [24,25,26]. At the same time, the presence of periodicity and the variation in phononic crystal parameters make it possible to artificially impose various acoustic properties even on isotropic materials.

This paper represents a theoretical study of two-dimensional phononic crystals made of fused silica. The distributions of all the main acoustic characteristics of practical importance in the creation of acousto-optic devices are obtained. The distributions of the inverse phase velocities of acoustic modes of a phononic crystal, the directions of their polarizations, and the acoustic energy walk-off angles are presented. Calculations of the acoustic characteristics are carried out by analyzing the phononic crystal band structure. The dispersion dependences of acoustic waves propagating in a phononic crystal are obtained by solving the elastic wave equation in its variational form, as described in [17]. The work presents the results of studies conducted to assess the possibility of using the acoustic properties of phononic crystals in creating acousto-optic devices.

## 2. Theoretical Model and Formulations

**Figure 2:**
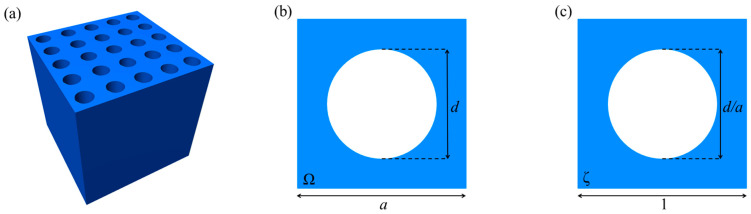
Model of a two-dimensional square-lattice phononic crystal: (**a**) three-dimensional physical model, (**b**) unit cell, and (**c**) unit cell, made dimensionless.

This paper analyzes a two-dimensional square-lattice phononic crystal based on fused silica. The model of the phononic crystal considered in the work is shown in Figure 2. The phononic crystal represents a sample of fused silica with periodically located cylindrical through holes filled with air (Figure 2a). It is considered an infinite, perfectly periodic medium.

The spatial periodicity of a phononic crystal is characterized by its unit cell, as shown in Figure 2b. The most important geometric parameters of the unit cell are the diameter of its cylindrical holes, d, and the lattice constant, a, which defines the length of the unit cell; the region Ω denotes the part of the unit cell taken up by fused silica.

Fused silica is chosen as a matrix material forming the phononic crystal, due to its wide availability and ease in manufacturing. In addition, fused silica is known for its low attenuation of acoustic waves [27]. Thus, when considering the problem of propagation of elastic waves in a phononic crystal, fused silica is considered an ideal medium and losses due to the propagation of acoustic waves are not considered. A possible approach to accounting for the viscoelastic properties of a phononic crystal, leading to the attenuation of acoustic waves, is presented in [28]. In our work, we also assume that the propagation of acoustic waves in a phononic crystal occurs only in the material of fused silica. The excitation and propagation of acoustic waves in cylindrical holes can be neglected due to the relatively low acoustic impedance of the air.

The theoretical model is based on the elastic wave equation of the form:

(1) $$\frac{\partial T_{ij}}{\partial x_{j}}+f_{i}=\rho \frac{\partial^{2}u_{i}}{\partial t^{2}},$$

where *u_i_* is an elastic wave displacement vector, *T_ij_* is a Cauchy stress tensor characterizing the reaction of the medium to the applied mechanical stress, *f_i_* is the bulk density of external forces, and ρ is the bulk density of the medium in which the elastic wave propagates. The indices take values of *i,j* = 1,2,3 and obey the Einstein convention.

The degree of deformation of the medium under the impact of an elastic wave propagating in it is characterized by the strain tensor *S_kl_*, which is defined as:

(2) $$S_{kl}=\frac{1}{2}\left(\frac{\partial u_{k}}{\partial x_{l}}+\frac{\partial u_{l}}{\partial x_{k}}\right).$$

In the limit of the small strains, the stress tensor *T_ij_* is proportional to the strain tensor *S_kl_* of the medium:

(3) $$T_{ij}=c_{ijkl}S_{kl},$$

where *c_ijkl_* is an elastic constants tensor of the medium. The latest equation represents Hooke’s law in the case of an anisotropic medium, in which stresses and strains are related linearly.

Given that the *c_ijkl_* tensor is symmetric with respect to the second pair of indices, in the absence of external forces to the bulk material, the wave equation (Equation (1)) can be written as follows:

(4) $$\frac{\partial }{\partial x_{j}}\left(c_{ijkl}\frac{\partial u_{k}\left(r,t\right)}{\partial x_{l}}\right)=\rho \frac{\partial^{2}u_{k}\left(r,t\right)}{\partial t^{2}}.$$

In the case of a phononic crystal, the elastic moduli *c_ijkl_*(*r*) and the bulk density ρ(*r*) are step functions of coordinates. Since the propagation of elastic waves occurs only in the material of fused silica, these functions take on the values of the corresponding constants of fused silica in the Ω region, and they are equal to zero in the region of cylindrical holes.

The spatial periodicity of a phononic crystal may be accounted for using Bloch’s theorem. In this case, the general solution of Equation (4) has the form of Bloch waves:

(5) $$u_{i}\left(r,t\right)={\tilde{u}}_{i}\left(r\right)\exp\left[i\left(\omega t-k\cdot r\right)\right],$$

where the amplitude *ũ_i_*(*r*) is a periodic function of coordinates, defined in the Ω region of the phononic crystal unit cell, and equal to zero in the region of cylindrical holes; *ω* = 2πf is the angular frequency of the elastic wave; and *k* is the wave vector of the elastic wave.

The vector *ũ*(*r*) sets the amplitude of the elastic wave and defines the direction of its polarization. The absolute value of the wave vector |*k*| = *ω*/V sets the phase velocity of acoustic waves propagating in a phononic crystal. Based on the axial symmetry of the unit cell, we limit the analysis to the wave vector in the form k = (k_x_, k_y_, 0).

The strain tensor of a periodic medium also, according to its definition (Equation (2)), takes the form of a Bloch wave:

(6) $$S_{ij}={\tilde{S}}_{ij}\left(\tilde{u}\right)\exp\left[i\left(\omega t-k\cdot r\right)\right],$$

where the amplitudes $\tilde{S}$ *_ij_* equals:

(7) $${\tilde{S}}_{ij}\left(\tilde{u}\right)=\frac{1}{2}\left[\left(\frac{\partial {\tilde{u}}_{i}}{\partial x_{j}}+\frac{\partial {\tilde{u}}_{j}}{\partial x_{i}}\right)-i\left(k_{i}{\tilde{u}}_{j}+k_{j}{\tilde{u}}_{i}\right)\right].$$

**Figure 3:**
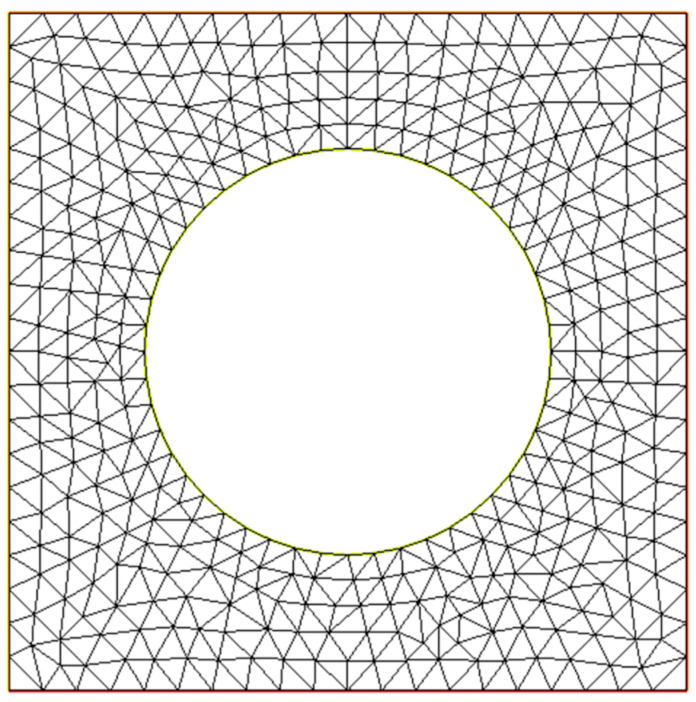
An example of the finite element method mesh used in calculation for a phononic crystal with *d/a = 0.6*.

The explicit form of solution (Equation (5)) allows obtaining the variational formulation of the wave equation (Equation (4)) for infinite periodic media. The resulting variational problem can be solved by the finite element method (FEM), which makes it possible to find the dispersion dependences of acoustic waves. The core idea of the method is to approximate a solution belonging to some infinite-dimensional function space (e.g., Sobolev space) by a linear combination of the fixed-basis vectors of a subspace of the space. The FEM approximation depends on the choice of a subspace and the associated basis. Usually, a space of piecewise polynomial functions is chosen as such a subspace. In our research, we used the Lagrange P_2_-elements as a basis. After choosing the basis functions, the FEM comes down to the optimization problem of minimizing the approximation error of some functional (given by a weak formulation of the original differential equation) on the FEM mesh. An example of the FEM mesh used in our calculcations for a phononic crystal with *d/a =* 0.6 is shown in Figure 3. According to [17], the described variational problem takes the following form:

(8) $$\int_{\Omega }{\tilde{S}}_{I}^{*}\left(v\right)c_{IJ}{\tilde{S}}_{J}\left(\tilde{u}\right)d\Omega =\omega^{2}\int_{\Omega }\rho \left(v^{*},\tilde{u}\right)d\Omega .$$

Here, *v*(*r*) is an arbitrary test function that belongs to the same functional space of finite elements as the solution *ũ*(*r*). Introduction of this function is necessary for solving the problem using the finite element method. In this case, the Voigt notation is used for tensor quantities, so the indices I ↔ (*ij*) and J ↔ (*kl*) take values from 1 to 6 according to the rule 1 ↔ (11), 2 ↔ (22), 3 ↔ (33), 4 ↔ (23), 5 ↔ (13), 6 ↔ (12). Considering the thermodynamic relations, the components of the six-dimensional deformation amplitudes vector are defined as $\tilde{S}$ *_I_* = (2 − δ*_ij_*) • $\tilde{S}$ *_ij_*, where δ*_ij_* is the Kronecker symbol.

The integral equation (Equation (8)) is a generalized eigenfunction and eigenvalue problem. The eigenvalue in this case is λ = ω^2^. The set of eigenvectors *ũ_λ_* determines the amplitudes and polarization directions of acoustic waves of frequency ω, which can propagate in a phononic crystal. According to Equation (7), the amplitude of the deformation vector is directly affected by the components of the wave vector. Thus, by changing the value of the wave vector within the first zone and solving the integral equation (Equation (8)) for each individual value of the wave vector k, one can obtain the dispersion relation ω(k).

To identify the main features of the solution to the integral equation (Equation (8)), it has to be brought to its dimensionless form. The following dimensionless quantities are introduced:

(9) $$\chi_{i}=\frac{x_{i}}{a},\;\;\xi_{i}=\frac{{\tilde{u}}_{i}}{a},\;\;\eta_{i}=\frac{v_{i}}{a},\;\;\;\kappa_{i}=k_{i}\cdot a,\;\;\rho =\rho^{\prime }\cdot \rho_{0},\;\;c_{IJ}=c_{IJ}^{'}\cdot c_{0},$$

where ρ′ and *c*′_IJ_ are dimensionless bulk density and elastic constants, respectively, carrying the numerical values of the corresponding physical constants of fused silica, and ρ_0_ and c_0_ are quantities that characterize the dimensions of those physical constants. In dimensionless quantities, the integral equation (Equation (8)) takes the form:

(10) $$\int_{\varsigma }S_{I}^{*}\left(\eta \right)c_{IJ}^{'}S_{J}\left(\xi \right)d\varsigma =\frac{\rho_{0}\omega^{2}a^{2}}{c_{0}}\int_{\varsigma }\rho^{\prime }\left(\eta^{*},\xi \right)d\varsigma ,$$

where the integration is carried out over the region ζ of the unit cell, which is made dimensionless, as shown in Figure 2c. The explicit form of the integral equation (Equation (10)) allows making several important observations.

First, the eigenvalues of this equation, and therefore also the eigenvalues of Equation (8), are not as dependent on the specific values of a and d as on their ratio *d/a*. Theoretically, the normalized hole diameter can be infinitely close to unity. However, the maximum *d/a* ratio considered in this work is 0.8. This choice pertains to the technological difficulties in creating phononic crystals, where the normalized hole diameter exceeds this value. It should be noted that increasing the diameter of the holes reduces the space taken up by the matrix material. This, in turn, leads to a decrease in the acousto-optic interaction region, which results in a lower diffraction efficiency. In addition, for a given geometry of a phononic crystal, that is, for a fixed region of integration ζ, the product of ω•a = const. Thus, a change in the unit cell constant *a* of a phononic crystal leads to the corresponding scaling of the dispersion dependences. Further in this work, for the sake of presentation clarity, we consider the solution of the integral equation (Equation (8)) in dimensional quantities. The unit cell constant *a* is considered equal to 10 μm.

## 3. The Method for Calculations of the Phononic Crystal Acoustic Characteristics

**Figure 4:**
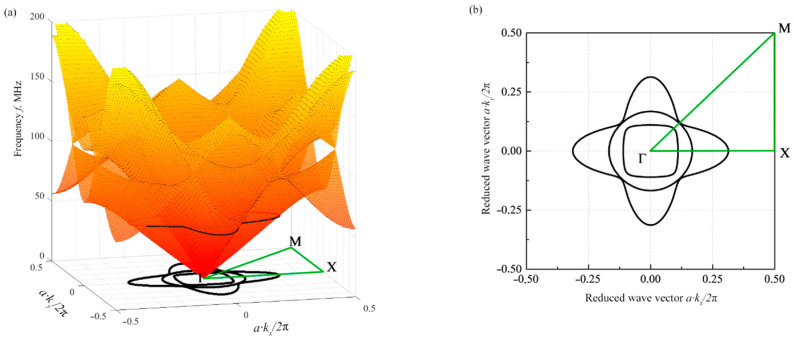
Band structure of a two-dimensional phononic crystal with a normalized hole diameter *d/a* = 0.8: (**a**) the first three dispersion surfaces corresponding to the three lowest acoustic modes in a phononic crystal and (**b**) projection of the dispersion surface contour lines onto the first Brillouin zone at an isofrequency *f*_0_ = 50 MHz.

A consistent solution of the generalized eigenfunction and eigenvalue problem (Equation (8)) for different values of the wave vector *k* makes it possible to obtain the dispersion dependence ω(k) of acoustic waves propagating in a phononic crystal. Figure 4 shows the band structure of a phononic crystal with a normalized hole diameter *d/a* = 0.8. Figure 4a shows the dispersion dependences calculated by the finite element method. Only the first three dispersion surfaces are displayed. They correspond to three different acoustic modes with a frequency of up to 200 MHz. Higher-order surfaces are above the indicated frequency. There are no absolute band gaps in the frequency range of up to 200 MHz and this crystal geometry, as evident from Figure 4a.

The dispersion surfaces of the first three acoustic modes of the phononic crystal originate from the center of the first Brillouin zone. In the lower frequency range, up to 50 MHz, these surfaces are mutually exclusive and monotonic and do not intersect. With higher frequencies exceeding 50 MHz, the surfaces are no longer monotonic. The emerging frequency regions with a negative slope may lead to a negative refraction effect. At frequencies above 100 MHz, the second and third surfaces begin to intersect. Such a complex structure is explained by the relatively high *d/a* ratio. It is also evident from Figure 4a that the dispersion surfaces are symmetric with respect to the first irreducible Brillouin zone Γ-X-M-Γ, which is explained by the symmetry of the unit cell.

The dispersion relation ω(**k**) connects the frequencies and wave vectors of acoustic waves that can propagate in a medium. By fixing the frequency at ω_0_ = 2πf_0_, it is possible to obtain sets of wavenumbers k_x_(f_0_) and k_y_(f_0_) of all acoustic waves of frequency f_0_ that can propagate in a phononic crystal. At a given ultrasound frequency *f*_0_, the wavenumbers k_x_ and k_y_ are proportional to the components of the inverse phase velocity of acoustic waves, since *k_x_* = 2π*f*_0_/*V_x_* and *k_y_* = 2π*f*_0_/*V_y_*. Thus, the line of the frequency contour *f*_0_ of the dispersion dependence defines the cross sections of the acoustic slowness surface S(φ) in the XY plane of the phononic crystal, where φ is the polar angle. In an acousto-optic device, the isofrequency *f*_0_ is the frequency of ultrasound excited by the piezoelectric transducer.

Figure 4a shows the contour lines of the isofrequency *f_0_* = 50 MHz, while the projection of those contour lines to the first Brillouin zone can be seen in Figure 4b. Similar to the dispersion surfaces, the isofrequency contour lines are symmetric with respect to the first irreducible Brillouin zone Γ-X-M-Γ. For isofrequencies *f_0_* < 50 MHz, the contour line of each individual acoustic mode is a closed curve. For isofrequencies *f_0_* > 50 MHz, where the dispersion surfaces become nonmonotonic, the contour lines become piecewise interval lines. The calculation of acoustic characteristics at these higher isofrequencies requires a separate analysis. This work presents the case in which the isofrequency is chosen to be *f_0_* = 50 MHz.

The spatial periodicity of a phononic crystal leads to anisotropy of the physical characteristics of acoustic waves propagating in a phononic crystal. In particular, the vectors of the phase and group velocities of acoustic waves become non-codirectional. The explicit form of the acoustic slowness curves S(φ) allows finding the angle ψ of the acoustic energy walk-off. The acoustic energy walk-off angle between the Pointing vector and the acoustic wave phase velocity, according to [6], can be found as:

(11) $$\psi =\tan^{-1}\left(\frac{1}{S}\frac{dS}{d\varphi }\right).$$

In acousto-optics, one of the most important characteristics of an acoustic wave is the direction of its polarization. The eigenvectors **ũ**_λ_(**r**) of the integral problem (Equation (8)) define the amplitudes and polarization directions of ω frequency acoustic waves, which can propagate in a phononic crystal. Since eigenvectors **ũ**_λ_(**r**) are periodic functions of coordinates, the polarization direction of each acoustic mode varies within the unit cell. Therefore, from a practical point of view, it seems necessary to carry out the calculation of the polarization vector **ũ**^0^ averaged over the unit cell region. The components of the averaged polarization vector can be obtained as:

(12) $${\tilde{u}}_{p}^{0}=\pm {\left(\frac{\int_{\Omega }{\left|{\tilde{u}}_{\lambda ,p}\right|}^{2}d\Omega }{\int_{\Omega }{\left|{\tilde{u}}_{\lambda }\right|}^{2}d\Omega }\right)}^{1/2},$$

where the index *p* = *x*,*y*,*z*.

According to Equation (12), the squared values of the integral equation (Equation (8)) solution components are averaged over the unit cell region of the phononic crystal and then normalized. However, only the squared values of the averaged polarization vector components can be obtained this way. The actual components ${\tilde{u}}_{p}^{0}$ of the averaged polarization vector **ũ**^0^ are defined to within an overall sign, as described in Equation (12). It is clear that in the limit *d/a* → 0, the averaged solution of Equation (8) should converge to that of solid isotropic fused silica. The requirement of the physical behavior in the mentioned limit makes it possible to choose the sign in Equation (12). We also assume that with a small change in the phononic crystal geometry, that is, with a small change in *d/a*, the change in the polarization vector direction is continuous. With this assumption, the signs of the components of the averaged polarization vector in Equation (12) can be determined for any values of the ratio *d/a*.

## 4. Results and Discussion

This section presents the results of calculating the main acoustic characteristics of a phononic crystal. The calculations are carried out according to the method described in the previous section. The values of the material constants of fused silica are chosen according to [6]. The elastic constants and density of fused silica are considered as *c*_11_ ≡ *c*_1_ = 7.85·10^10^ N/m^2^, *c*_12_ ≡ *c*_6_ = 1.61·10^10^ N/m^2^, and ρ = 2203 kg/m^3^.

### 4.1. Inverse Phase Velocity Distributions

Before moving on to the various geometries of a phononic crystal, we present the slowness curves of solid isotropic fused silica that can be taken as a reference material. It is well known that in the isotropic case, there are two transverse and one longitudinal acoustic mode propagating in the medium. The polarizations of these modes are mutually orthogonal. One of the ways to obtain the phase velocities of these modes is by solving the Christoffel equation (Equation (6)). The phase velocities of the transverse and longitudinal acoustic modes are:

(13) $$V_{⊥}={\left(\frac{c_{11}-c_{12}}{2\rho }\right)}^{1/2}=\;\;3763\;m/s,\;V_{∥}={\left(\frac{c_{11}}{\rho }\right)}^{1/2}=\;5969\;m/s.$$

**Figure 5:**
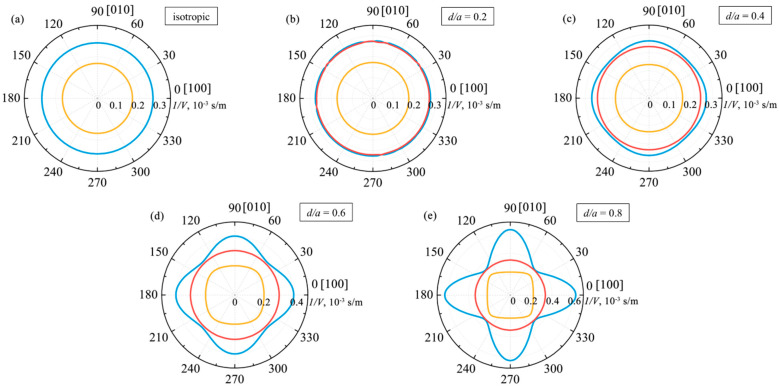
(**a**) Inverse phase velocity curves in the XY plane in an isotropic material of fused silica. Shown in (**b**–**e**) are inverse phase velocity curves of a phononic crystal with the normalized hole diameter: (**b**) d/a = 0.2; (**c**) d/a = 0.4; (**d**) d/a = 0.6; and (**e**) d/a = 0.8.

In the case of solid isotropic fused silica, the cross sections of the acoustic slowness surfaces are a pair of circles, as shown in Figure 5a. The two transverse shear acoustic modes are shown in blue; the longitudinal acoustic mode is shown in orange.

Figure 5b shows the cross sections of the slowness surface of a phononic crystal with the normalized hole diameter *d/a* = 0.2. Clearly, the introduction of a spatially periodic inhomogeneity into the material of solid isotropic fused silica leads to the gradual elimination of the degeneracy of transverse acoustic modes, while increasing the normalized hole diameter to *d/a* = 0.4, as shown in Figure 5c, results in the complete removal of the degeneracy. Thus, transverse modes of different polarization directions propagate at different speeds in a phononic crystal. In Figure 5b,c, this is reflected by the appearance of a red curve.

A further increase in the normalized hole diameter leads to notable anisotropy of the phase velocity. One can see that in the case of Figure 5d with the normalized hole diameter *d/a* = 0.6, the longitudinal mode becomes anisotropic, just as well as the slow shear mode. However, the fast shear mode of the phononic crystal shown in red in Figure 5d remains isotropic.

The degree of anisotropy of the acoustic mode phase velocity is characterized by the anisotropy coefficient, which is defined as:

(14) $$\chi \;=\;\left({\frac{V_{max}}{V_{min}}}^{2}\right),$$

where $V_{max}$ and $V_{min}$ are the maximum and minimum values of the phase velocity of an individual acoustic mode, respectively. The anisotropy coefficients of the phononic crystal acoustic modes, as well as their minimum and maximum phase velocities, are presented in Table 1.

The phononic crystal acoustic slowness curves with the normalized hole diameter *d/a* = 0.8 are of greatest interest (Figure 5e). It is clear that the slow shear acoustic mode has an extremely strong anisotropy of phase velocity. The anisotropy coefficient of that mode is χ = 3.49. The longitudinal acoustic mode is also anisotropic, with an anisotropy coefficient χ = 1.46. However, the fast shear mode shown in red in Figure 5e remains isotropic even at such a large value of the *d/a* ratio. Further analysis shows that this is due to the fact that the shear mode of interest is polarized along the cylindrical holes (*Z* axis).

The results presented in Figure 5 make it possible to observe the dynamics of changes in the distributions of the inverse phase velocities of acoustic modes, depending on the normalized hole diameter *d/a*. Clearly, an increase in the normalized diameter leads to an increase in the anisotropy of the phase velocities. At the same time, as evident from Table 1, an increase in the normalized hole diameter leads to a decrease in the phase velocities of acoustic modes. The latter is also true for the fast shear mode of a phononic crystal, which is isotropic. Thus, by changing the normalized diameter of the cylindrical holes, it seems possible to impose the necessary distributions and numerical values of the phase velocities of the acoustic modes on the phononic crystal. The obtained results are in qualitative agreement with the slowness curves for the two-dimensional metamaterial based on rutile [29].

### 4.2. The Distributions of Walk-Off Angles

The explicit form of the acoustic slowness curves makes it possible to calculate the walk-off angle according to Equation (11). For a fixed direction of acoustic wave propagation, the walk-off angle defines the angle between the normal to the slowness curve and the direction of wave propagation. Since in the case of isotropic fused silica, the slowness curves of acoustic modes are a pair of circles (Figure 5a), the walk-off angles of these modes are exactly zero for all directions of their propagation. In the case of small values of the normalized hole diameter, the inverse phase velocity curves are of nearly round shape and the acoustic walk-off angle is close to zero. Thus, in a phononic crystal with the normalized hole diameter *d/a*= 0.2, the walk-off angles of acoustic modes do not exceed several degrees.

**Figure 6:**
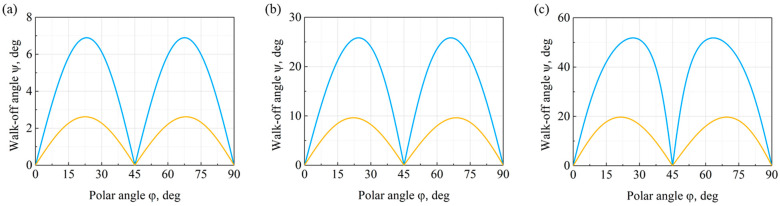
Distribution of acoustic energy walk-off angles in the XY plane of a phononic crystal with a normalized hole diameter: (**a**) *d/a* = 0.4; (**b**) *d/a* = 0.6; and (**c**) *d/a* = 0.8.

Figure 6 shows the distributions of the walk-off angles ψ (φ) of acoustic modes, depending on the direction of their propagation in phononic crystals of various *d/a* ratios. The presented walk-off angle distributions of the slow shear and longitudinal acoustic modes correspond to the slowness curves shown in Figure 5c,d. For the fast shear mode, the walk-off angle ψ (φ) distributions are not shown, because the mode is isotropic and its energy walk-off angle equals zero for all propagation directions.

With a relatively small normalized hole diameter of *d/a* = 0.4, the maximum walk-off angle of a phononic crystal acoustic mode does not exceed 7°, as is evident from Figure 6a. According to Figure 6b, a further increase in the normalized hole diameter to *d/a* = 0.6 leads to a sharp increase in the acoustic walk-off angle. In particular, the maximum value of the walk-off angle of the slow shear mode exceeds 25°. The maximum values of the energy walk-off angles, ψ _max_, of acoustic modes in phononic crystals with different normalized hole diameters are presented in Table 2, as well as the propagation directions of the modes, φ *, leading to the maximum walk-off angle.

The walk-off angle distributions of acoustic modes shown in Figure 6c are of practical interest. The results suggest that the maximum walk-off angle of the slow shear acoustic mode exceeds 50°. It should be noted that this value is unusually high, and there are only a few monocrystalline structures currently known that exhibit such a large walk-off of acoustic energy. Thus, changing the geometry of a phononic crystal makes it possible to control the energy walk-off angles over a wide range of values.

### 4.3. The Polarizations of Acoustic Modes

One way of finding the polarization directions of the acoustic modes in isotropic solid fused silica is by solving the Christoffel equation. It has been established that due to the symmetry of the Christoffel tensor, the polarizations of acoustic modes in solid fused silica are mutually orthogonal for any direction of acoustic mode propagation [6]. Calculations indicate that one of the purely shear acoustic modes shown in Figure 5a is polarized in the XY plane of the figure. The other purely shear acoustic mode is polarized along the *Z* axis. The polarization of the purely longitudinal acoustic mode lies in the XY plane, and it is orthogonal to the polarization directions of the shear acoustic modes.

**Figure 7:**
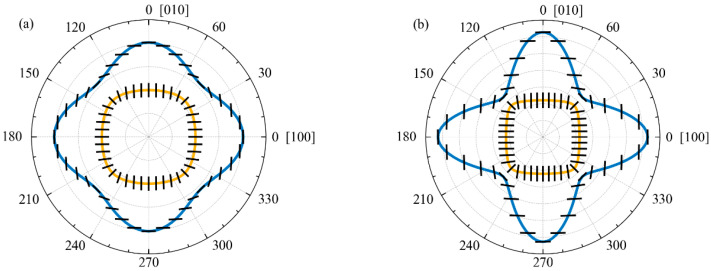
Distribution of the averaged polarization vector of acoustic modes in a phononic crystal with a normalized hole diameter: (**a**) *d/a* = 0.6 and (**b**) *d/a* = 0.8.

Figure 7 presents the distributions of the direction of the averaged polarization vector **ũ**^0^(φ) of acoustic modes, depending on their propagation direction, featuring two different geometries of a phononic crystal. The averaged polarization vector is calculated using the method described in the previous section. The results show that the fast shear mode is polarized along the *Z* axis of the cylindrical holes of the phononic crystal. The slow shear acoustic mode appears purely transverse for propagation directions φ=πn/4, n∈ℕ in the XY plane. The longitudinal acoustic mode of a phononic crystal is purely longitudinal when propagating along the indicated directions. This property of the averaged polarization vector is associated with the symmetry of the phononic crystal unit cell.

The degree of polarization of the acoustic mode can be characterized by the angle γ^0^(φ) between the averaged polarization vector **ũ**^0^(φ) and the direction of the mode φ propagation. Same as the averaged polarization vector, this angle is a function of the acoustic wave propagation direction. By definition of the corresponding terms, for quasi-shear waves, the value of this angle lies in the region 45° ≤ γ^0^ < 90°; for quasi-longitudinal waves, it lies in the region 0° < γ^0^ ≤ 45°. The polarization angle γ^0^ of the fast shear isotropic mode of a phononic crystal is 90° for all directions of wave propagation. This mode is purely shear, and it is polarized along the *Z* axis of the cylindrical holes for all phononic crystal geometries.

In accordance with Figure 7a, for the phononic crystal geometry with a normalized hole diameter *d/a* = 0.6, the polarization angle of the slow shear mode of a phononic crystal belongs to the 69.9° ≤ γ^0^ ≤ 90° range. The polarization angle of the longitudinal mode takes the values 0° ≤ γ^0^ ≤ 20.1°. For the phononic crystal geometry in which *d/a* = 0.8, the polarization angle of the slow shear mode lies in the range of 61.6° ≤ γ^0^ ≤ 90°, as seen in Figure 7b. For the longitudinal mode of a phononic crystal, the range is 0° ≤ γ^0^ ≤ 28.4°. Thus, the anisotropic acoustic modes of a phononic crystal are quasi-transverse and quasi-longitudinal. The transformation of the acoustic mode from quasi-transverse to quasi-longitudinal and vice versa is impossible in a phononic crystal. The limitation is associated with the symmetry of the phononic crystal unit cell, as well as with the assumption that the averaged polarization vector **ũ**^0^(φ) is continuous.

The calculations of averaged polarization vectors help to trace an unusual characteristic of their behavior. It is evident from the results presented in Figure 7 that the averaged polarization vectors of the phononic crystal acoustic modes are mutually orthogonal for any directions of wave propagation at any values of the normalized hole diameter *d/a*. This property is typical for continuous homogeneous media; however, it is not universal for periodic structures in general.

In the case of homogeneous media, the mutual orthogonality of acoustic modes arises from the symmetry of the Christoffel tensor. The kernel of the integral equation (Equation (8)), which makes it possible to identify the acoustic modes of a phononic crystal, is also symmetric with respect to the solution **ũ***_λ_*. However, this symmetry is not the reason for the orthogonality of the polarization vectors of the phononic crystal acoustic modes, since for any given propagation direction φ, the solution **ũ***_λ_* is sought at various wavenumbers *k_x_* and *k_y_*. The eigenvalue of the integral equation (Equation (8)) for different acoustic modes matches because these modes are obtained by isocontouring the dispersion surfaces of a fixed isofrequency *f_0_*. However, when searching for the averaged polarization vectors of acoustic modes, different integral equations with different kernels are solved. Thus, in the low-frequency region of excited acoustic modes, up to 50 MHz, a phononic crystal effectively behaves as a homogeneous anisotropic medium in the sense that there are exactly three acoustic modes in it, and their mean polarizations are mutually orthogonal.

## 5. Conclusions

This work considers the acoustic properties of two-dimensional square-lattice phononic crystals based on fused silica. Distributions of the main characteristics of the phononic crystal acoustic modes are calculated, such as inverse phase velocities, directions of their polarizations, and acoustic energy walk-off angles. It is established that the introduction of spatial periodicity into an initially isotropic material of fused silica leads to strong anisotropy of the acoustic properties. Thus, the energy walk-off angle of the slow shear acoustic mode reaches ψ = 51.8° when the mode propagates along certain directions. It is shown that a change in the phononic crystal geometry makes it possible to control both the spatial distributions and the numerical values of its acoustic characteristics. In particular, the geometry of a phononic crystal is presented, for which the minimum value of the slow shear acoustic mode phase velocity is V = 1594 m/s. This is several times smaller than the phase velocity value V_⊥_ = 3763 m/s of the shear acoustic mode of solid isotropic fused silica. It is demonstrated that under certain conditions, exactly three acoustic modes propagate in a phononic crystal. In this case, the averaged polarization vectors of acoustic modes are mutually orthogonal for any directions of wave propagation. Thus, under certain conditions, a phononic crystal effectively behaves as a homogeneous bulk anisotropic medium.

From the point of view of acoustic properties, phononic crystals satisfy all the necessary requirements for materials in the design of acousto-optic devices. Therefore, the main conclusion of the work is that phononic crystals can be recommended for use in acousto-optic devices, namely filters, deflectors, and modulators, where sound velocity control is required. More practical applications include the use in acoustic filters and delay lines.

## Figures and Tables

**Table 1:** Phase velocities of acoustic modes and their anisotropy coefficients for phononic crystals with various normalized hole diameters.

*d/a*	Slow Shear Wave		Isotropic Wave	Longitudinal Wave	
	$V_{min},\;m/s$	$V_{max},\;m/s$	$V_{iso},\;m/s$	$V_{min},\;m/s$	$V_{max},\;m/s$
0.2	3707	3708	3709	5808	5816
	χ ≈ 1			χ ≈ 1	
0.4	3191	3392	3542	5315	5438
	χ = 1.13			χ = 1.05	
0.6	2481	3174	3310	4590	5007
	χ = 1.64			χ = 1.19	
0.8	1594	2976	3004	3759	4536
	χ = 3.49			χ = 1.46	

**Table 2:** Maximum values of the energy walk-off angles of acoustic modes in phononic crystals with various normalized hole diameters.

*d/a*	Slow Shear Mode		Longitudinal Mode	
	ψ _max_, °	φ *, °	χ_max_, °	ψ *, °
0.4	6.9	23.3	2.62	22.7
0.6	25.8	24.6	9.6	22.2
0.8	51.8	27.4	19.7	21.5
